# Structural Modeling and Biochemical Characterization of Recombinant KPN_02809, a Zinc-Dependent Metalloprotease from *Klebsiella pneumoniae* MGH 78578

**DOI:** 10.3390/ijms13010901

**Published:** 2012-01-16

**Authors:** Mun Teng Wong, Sy Bing Choi, Chee Sian Kuan, Siang Ling Chua, Chiat Han Chang, Yahaya Mohd Normi, Wei Cun See Too, Habibah A. Wahab, Ling Ling Few

**Affiliations:** 1 School of Health Sciences, Health Campus, Universiti Sains Malaysia, Kubang Kerian 16150, Kelantan, Malaysia; E-Mails: wendywong84@gmail.com (M.T.W.); kcs10_psk046@student.usm.my (C.S.K.); siang_ling85@yahoo.com (S.L.C.); chiathan@gmail.com (C.H.C.); stweicun@kb.usm.my (W.C.S.T.); 2 Pharmaceutical Design and Simulation (PhDS) Laboratory, School of Pharmaceutical Sciences, Universiti Sains Malaysia, Minden 11800, Pulau Pinang, Malaysia; E-Mail: sybing@gmail.com; 3 Department of Cell and Molecular Biology, Faculty of Biotechnology and Biomolecular Sciences, Universiti Putra Malaysia, Serdang 43400, Selangor, Malaysia; E-Mail: normi@biotech.upm.edu.my

**Keywords:** *Klebsiella pneumoniae* MGH 78578, hypothetical protein, homology modeling, molecular docking simulation, metalloprotease, metalloprotease inhibitors, *ypfJ* gene

## Abstract

*Klebsiella pneumoniae* is a Gram-negative, cylindrical rod shaped opportunistic pathogen that is found in the environment as well as existing as a normal flora in mammalian mucosal surfaces such as the mouth, skin, and intestines. Clinically it is the most important member of the family of Enterobacteriaceae that causes neonatal sepsis and nosocomial infections. In this work, a combination of protein sequence analysis, structural modeling and molecular docking simulation approaches were employed to provide an understanding of the possible functions and characteristics of a hypothetical protein (KPN_02809) from *K. pneumoniae* MGH 78578. The computational analyses showed that this protein was a metalloprotease with zinc binding motif, HEXXH. To verify this result, a *ypfJ* gene which encodes for this hypothetical protein was cloned from *K. pneumoniae* MGH 78578 and the protein was overexpressed in *Escherichia coli* BL21 (DE3). The purified protein was about 32 kDa and showed maximum protease activity at 30 °C and pH 8.0. The enzyme activity was inhibited by metalloprotease inhibitors such as EDTA, 1,10-phenanthroline and reducing agent, 1,4-dithiothreitol (DTT). Each molecule of KPN_02809 protein was also shown to bind one zinc ion. Hence, for the first time, we experimentally confirmed that KPN_02809 is an active enzyme with zinc metalloprotease activity.

## 1. Introduction

*Klebsiella* was first identified as a cause of pneumonia in 1882 by a pathologist Karl Friedlander [1]. *Klebsiella pneumoniae* is a Gram negative; rod shaped and encapsulated bacterium of the family Enterobacteriaceae, which normally inhabits the animal and human intestinal tract [2]. It is an opportunistic pathogen which causes many nosocomial infections such as pneumonia, urinary tract infection and septicemia, primarily on immunocompromised persons [3]. In Malaysia, it was reported to be present in 32% out of 78 street food samples from different states [3]. The incidence of community acquired pneumonia attributed to *K. pneumoniae* decreased over the year [3], however the mortality rate remains significant. This is due to the evolving multi-drug resistant *K. pneumoniae* strains [4] and other underlying diseases that tend to be aggressively present in the affected patient. *K. pneumoniae* was always treated by antibiotics, but the emergences of antibiotic resistant *K. pneumoniae* further increase the need to understand the bacteria-host interaction, host defense mechanism and also the cellular mechanism of the bacterium itself.

*K. pneumoniae* strain MGH 78578 is one of the strains that show high level of resistance to multiple antimicrobial agents including ampicillin, oxacillin, kanamycin, and chloramphenicol [5]. This strain was originally isolated from the sputum of a male patient in 1994 [5] and its genome has been sequenced by the Genome Sequencing Center of Washington University in Saint Louis in 2007. It was estimated that 20% of the total predicted open reading frames (ORFs) in the genome encode for hypothetical proteins, whose expressions and functions have not been proven experimentally. One of the hypothetical proteins is KPN_02809 which is encoded by the *ypfJ* gene.

The result of sequence similarities annotation by Uniprot [6] revealed that it belongs to a Zn metalloprotease family. Zinc metalloproteases catalyze peptide bond hydrolysis in a protein or peptide substrate. They contain divalent metal ions on their active sites; activate the water molecule as the direct attacking species on the peptide bond. Analysis of their sequences showed that zinc metalloproteases have the metal ion binding site, HEXXH, where X is any amino acid. The two histidine residues together with another residue (different among metalloprotease groups) at the active site are involved in the zinc binding [5]. Metalloprotease, the most diverse of the six main types of proteases, has drawn much of our interest as it plays an important role in host-pathogen interactions by promoting enteropathogenicity, vascular permeability, host tissue damage and cytotoxicity [7]. Metalloproteases expressed by pathogens such as *Legionella pneumophila*, *Vibrio cholerae* and *Plasmodium vivax* involve in pathogenesis of the disease by degrading a wide range of host molecules [8–10].

Despite its predicted function as a metalloprotease, the protease activity of KPN_02809 has never been experimentally confirmed and thus, it is still being designated as a hypothetical metalloprotease. This *ypfJ* gene product has never been investigated experimentally. Most of the proteases contain HEXXH site, however there are certain proteins with the HEXXH site that do not possess the protease activity [11]. Hence, in this work, besides presenting results from computational approaches to model the structure of this hypothetical protein in order to elucidate its function, we also report the cloning and expression of the open reading frame of *ypfJ* gene that encodes for this hypothetical protein. Characterization of the purified recombinant protein supported the results of the bioinformatics approaches.

## 2. Results and Discussion

### 2.1. Structural Modeling and Analysis of KPN_02809

Preliminary sequence analysis with a simple BLAST search against non-redundant (NR) database showed that *ypfJ* gene product, KPN_02809 belongs to a Zn metalloprotease family. It shared more than 90% sequence identity with other metalloprotease sequences in the NR database. However, when the BLAST search was performed against PDB, no particular structure was found with a good E-value. 11 structures were identified with the hits below E-value threshold. All of them shared low sequence identity, ~32% within a small coverage in the sequence and they were not from the Zn metalloprotease family. With such low sequence identity, we therefore decided to adopt the fold recognition approach for identifying the potential template from the Phyre [12] and InterproScan [13] servers for structural modeling.

**Figure 1:**
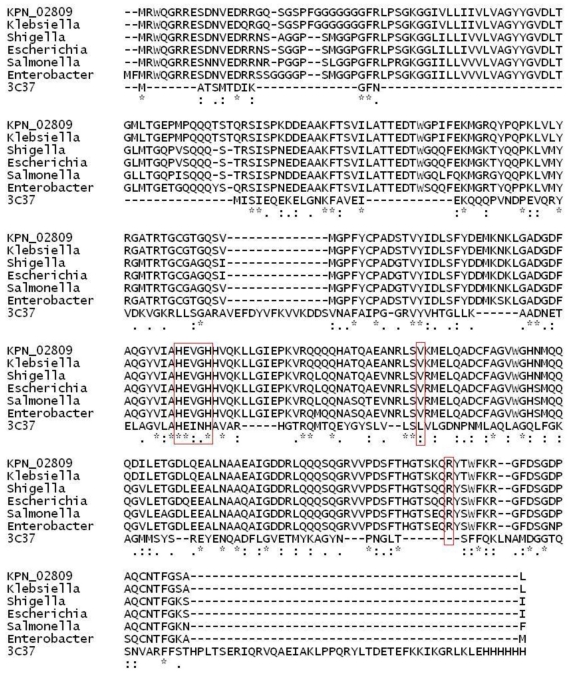
Multiple sequence alignment of KPN_02809 with related sequences from bacteria in Enterobactericea family. Conserved motif HEXXH and two important residues (Val-204 and Arg-268 according to KPN_02809 sequence) are highlighted in red.

Phyre [12] analysis identified 3C37 as the best template with a 98% estimated precision. 3C37 is an X-ray structure of a 253 amino acid putative M48 family Zn-dependent peptidase from *Geobacter sulfurreducens*. All the hits by InterproScan are known as M48 peptidase with almost 100% identity, however there was no known solved structure among them. Results from both Phyre and InterproScan indicated that KPN_02809 shared structural features with M48 zinc peptidase domain and 3C37 was selected as the template for model building. Figure 1 shows the multiple sequence alignment of KPN_02809 with top five hits from BLAST search and 3C37.

**Figure 2:**
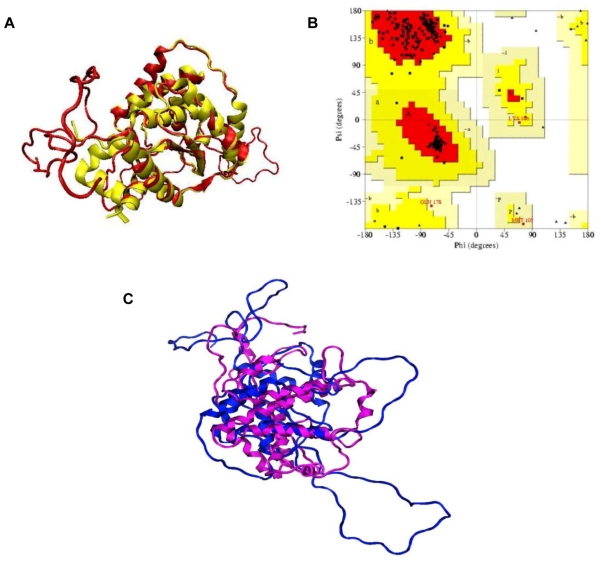
Structural model of KPN_02809. (**A**) Superposition of the built model of KPN_02809 (red) with template from protein data bank with PDB Code 3C37 (yellow); (**B**) Ramachandran plot analysis of KPN_02809 model. No residue was found in the disallowed region; (**C**) Superimposition of models by MODELLER (blue) and I-TASSER (purple) with RMSD of 16.81 Å. Structure built with MODELLER is more extended compared to I-TASSER model. However, the secondary structures in the conserved region for both models are very similar.

The best DOPE scoring model shown in Figure 2A was selected and subjected to Ramachandran plot analysis for the validation of the structure quality. The root-mean square deviation of the built model is 1.5 Å with the sequence identity of 11%. The core region is well overlapped with each other. The loops were built for sequences with no sequence similarity with the template. The core region where the unique metalloprotease HEXXH motif was located folded firmly in the center of the structure. Ramachandran plot validation of the quality of the structure (Figure 2B) showed that 92.9% of the total residues fell within the most favorable regions. None of the residue fell within the disallowed region. Alternatively, I-TASSER was also used to predict the structure of KPN_02809. Out of eight potential templates identified by I-TASSER, 3C37 was the only Zn-dependent peptidase with one of the highest sequence identity score. Ramachandran plot of the model predicted by I-TASSER showed that 84.9% of the total residues fell in the most favorable region while 4.6% of total residues fell in generously allowed and disallowed regions. Superimposition of structures modeled by MODELLER and I-TASSER is shown in Figure 2C. Majority of the secondary structures in the conserved region are preserved in both models although the RMSD is high (16.81 Å). Variations at the extended loops on both modeled structures are the major contributors to the high RMSD value. Model built with MODELLER was used in our subsequent simulations due to its higher quality, as shown by PROCHECK results.

Although the sequence identity between KPN_02809 and 3C37 was only 11%, however, the 11% identical sequence fell in the highly conserved domain of Zn-peptidases. Furthermore, our built structure also showed that the conserved sequences possessed the same secondary structure topology which made up the core region of this family of enzymes. The core region consists of eight strands of beta sheet sandwiched by six alpha helices. This unique topology is the trademark of Zn metalloprotease and it is also highly conserved as indicated by the ProBiS result (Figure 2C, left panel). In the KPN_02809 model structure, HEXXH is located very close to the Zn^2+^. In addition, two conserved residues indicated in the sequence alignment (Figure 1), namely Val204 and Arg268 are also located within 6 Å of the Zn^2+^ ion. Arg268 contributes to polar environment which stabilizes the substrate that is interacting with zinc ion [14]. The role of Val204 is not clear, it might be important for the structure of active site as it is highly conserved and located in close proximity to the zinc ion. The HEXXH sequence motif found in KPN_02809 is believed to be responsible for the Zn chelation [14]. In our built model, Zn^2+^ ion is located around the conserved core region and surrounded by two histidine residues from the HEXXH sequence motif. These histidine residues are responsible for the Zn^2+^ valent chelation which is important for the catalytic activity of KPN_02809 as a metalloprotease. The importance of the His in this motif had been studied in the Ste24p from yeast [9], another highly conserved metalloprotease. Result from the study showed that mutation of the conserved residue in this motif resulted in the lost of protein function [9]. Our subsequent experimental results proved that KPN_02809 is a Zn-dependent protease.

### 2.2. Molecular Docking Simulation

**Figure 3:**
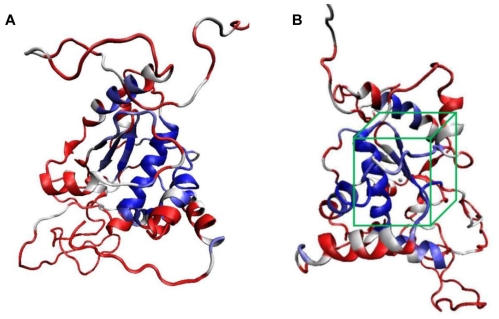
Structural conservation analysis by ProBis. (**A**) Conservation scoring of the KPN_02809 model by ProBiS. The highly conserved core region is represented in blue and white; (**B**) KPN_02809 predicted Zn binding site with the grid box (green) is centered on the Zn binding site.

Prior to the inhibitors docking simulation, identification of Zn binding site at KPN_02809 is necessary in order to define the grid centre for docking simulation. A total of 40 similar structures were selected in ProBis and all of them were aligned structurally using a local surface structural alignment approach. Unsurprisingly, 3C37 was one of the top similar structures that were selected by ProBis search. This correlates with the fold recognition result earlier by Phyre. It was found that the predicted Zn binding site, HEXXH, is conserved in the core center of the entire structure (Figure 3B). Hence, the grid center for inhibitors docking simulation is located at this core center. For the docking result, the most populated and the lowest binding free energy conformation was selected.

**Figure 4:**
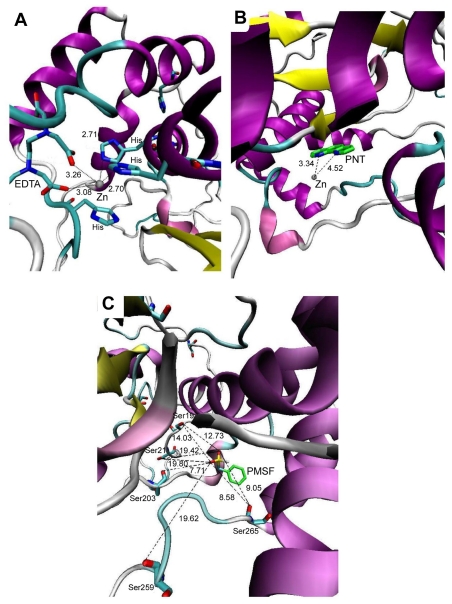
Molecular docking with selected protease inhibitors. (**A**) Docking of EDTA on the predicted Zn binding site. The docked EDTA was located close to the Zn^2+^ and the possibility of hydrogen bond formation is high; (**B**) Docking of PNT showing distances from Zn^2+^ that allows complex formation; (**C**) Docking of PMSF showing distances from serine residues surrounding the catalytic core. The distances are too far apart for hydrogen bond interactions. All distances are in angstrom (Å).

Common protease inhibitors such as EDTA, 1,10-phenanthroline (PNT) and PMSF were selected to probe the functionality of KPN_02809 as metal-dependent protease using molecular dynamic simulation. EDTA was found to bind stably in the binding pocket with a low free binding energy of −11.08 kcal/mol. Based on the docking result, the chelating of EDTA to Zn^2+^ exhibited a significant binding energy. EDTA was located close to the Zn^2+^ and within the hydrogen bonding cut-off (<3.5 Å) (Figure 4A). Thus, there is a possibility that the hydrogen bonds might form and suggests that the interaction of EDTA will inhibit the catalytic activity of KPN_02809. Docking simulation of PNT, another metal chelator, showed a distance of 3.34 Å and 4.52 Å from Zn^2+^ with a free binding energy of −7.29 kcal/mol (Figure 4B). The results supported the possibility of PNT forming a complex with the divalent zinc ion on KPN_02809. On the contrary, the docking simulation with PMSF, a serine protease inhibitor, showed that the distance between PMSF and the nearest serine residue was about 7.71 Å (free binding energy of 6.50 kcal/mol), which would not allow any hydrogen bond interaction (Figure 4C). The predicted inhibitory effects of EDTA and PNT towards the KPN_02809 protease activity were later proven experimentally in this work.

### 2.3. Detection of ypfJ mRNA Expression in K. pneumoniae MGH 78578

**Figure 5:**
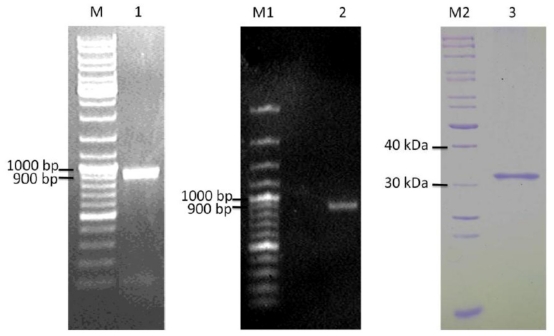
Cloning and purification of KPN_02809. Lane 1: Confirmation of KPN_02809 RNA expression by RT-PCR; Lane 2: PCR amplification of KPN_02809 ORF from *Klebsiella pneumoniae* MGH 78578 genomic DNA; Lane 3: Purified recombinant KPN_02809 protein. M: GeneRuler DNA ladder mix, M1: GeneRuler 100bp DNA ladder and M2: Unstained protein ladder from Fermentas.

The *ypfJ* mRNA was detected by RT-PCR as shown in Figure 5 (lane 1). A PCR product corresponding to the size of *ypfJ* ORF (876 bp) was successfully amplified from *K. pneumoniae* cDNA. The result confirms that *ypfJ* is not a pseudogene. This gene is readily transcribed into mRNA from the genome of *K. pneumoniae* in its normal growth condition which also indicated that the hypothetical protein KPN_02809 might be expressed in the *K. pneumoniae* in the same growth condition.

### 2.4. Cloning and Heterologous Expression of ypfJ Open Reading Frame in E. coli

*ypfJ* ORF with a size of 876 bp was amplified from *K. pneumoniae* MGH 78578 genomic DNA (Figure 5, lane 2) by PCR and it was cloned into pGEX-RB vector for the expression of KPN_02809 as a GST-tagged protein in *E. coli*. The optimal induction time and temperature were 2 hr and 25 °C. More than 80% of the GST-tagged KPN_02809 was present as soluble fraction under the purification procedure used in this study. The GST tag was removed in the final step and the typical yield of purified KPN_02809 (Figure 5, lane 3) per liter culture was around five milligrams. The size of the protein was about 32 kDa.

### 2.5. Proteolytic Activity of Purified Wild-type and Mutants K. pneumoniae KPN_02809

**Figure 6:**
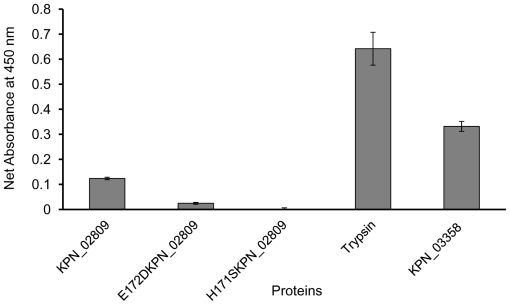
Proteolytic activities of wild-type and mutant KPN_02809. Activity was measured at 30 °C, pH 8 for 60 minutes. The reaction was initiated by adding 20 μg of enzyme protein into a microplate well containing 100 μg of succinylated casein. After incubation, 50 μL of TNBSA solution were added and incubated for an additional 30 minutes. The absorbance at 450 nm was determined and the proteolytic activity was represented by the change in absorbance (ΔA_450nm_), which was calculated by subtracting the absorbance of the blank. TPCK-Trypsin provided in the QuantiCleave Protease Assay kit and KPN_03358 were included as positive controls and for comparison.

**Figure 7:**
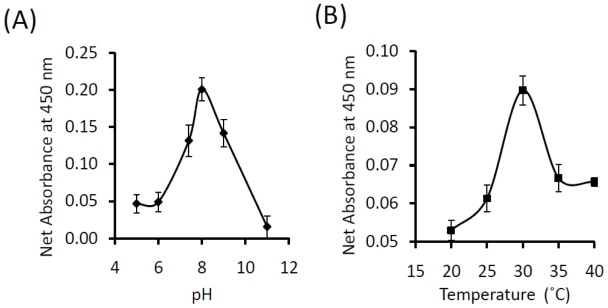
Effect of pH and temperature on the protease activity of KPN_02809. (**A**) The experiments were performed at 30 °C with succinylated casein as the substrate for 60 min; (**B**) The experiments were carried out at different temperatures with succinylated casein as the substrate for 60 minutes at pH 8. All values were reported as the means and standard deviations of three independent experiments.

Proteolytic activities of purified wild-type KPN_02809 and its HEXXH motif mutants (His171 to Ser and Glu172 to Asp) were determined by casein hydrolysis assay. TPCK-Trypsin provided in the QuantiCleave Protease Assay kit and KPN_03358, another zinc-dependent protease isolated from *K. pneumoniae* [15] were also included in the assay for comparison. As shown in Figure 6, the proteolytic activity of KPN_02809 was about 40% of KPN_03358 and 20% of trypsin. Mutations in the HEXXH motif resulted in significant loss of proteolytic activity, especially for H171S mutant, the activity was almost undetectable. The results also rule out the possibility that KPN_02809 activity was contributed by *E. coli* protease contamination. Wild-type KPN_02809 showed optimum proteolysis at 30 °C and pH 8 (Figure 7A and B). The results confirmed for the first time, that KPN_02809 is an active protease.

**Figure 8:**
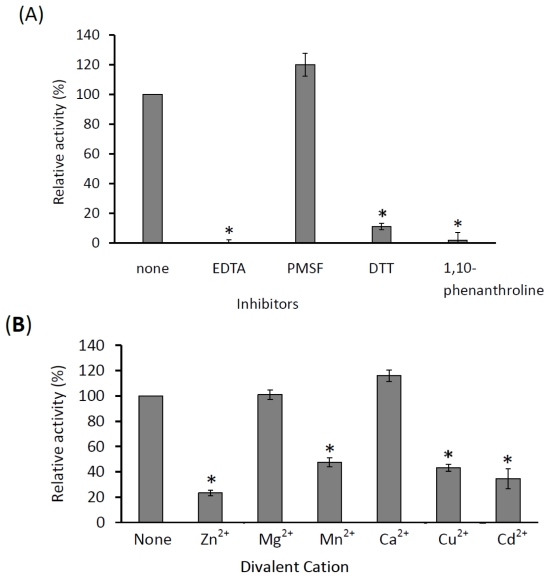
Effect of inhibitors and divalent cations on the activity of KPN_02809 protein. (**A**) The protein was preincubated with 1 mM of the potential inhibitors for 1 hour at room temperature before the enzyme activity assay at standard conditions was carried out; (**B**) 1 mM of different cations was added into the assay mixture and activity was determined at 30 °C. The activities of the protein with inhibitors were compared with the control. All the values reported represent the means and standard deviations of three independent measurements. * indicates significant difference at *p* ≤ 0.05.

A series of inhibitors were tested on the enzyme activity with succinylated casein as the substrate to determine the classification of this protease. Generally, proteases can be grouped as serine protease, threonine protease, cysteine protease or metalloprotease [16]. Our result showed that KPN_02809 was highly sensitive to EDTA (Figure 8A) which is a metal chelator as predicted by the molecular docking simulation. Besides EDTA, KPN_02809 protease activity was also inhibited by 1,10-phenanthroline and DTT (Figure 8A), which are known metalloprotease inhibitors [16]. Metallopeptidase form a very heterogenous family, with each of them widely different in sensitivities and specificities to inhibitors [5]. A diagnostic feature of metallopeptidase is the chelating agents such as 1,10-phenanthroline and EDTA, which could inhibit its enzymatic activity [10]. DTT is a strong reducing agent. Since there are four cysteine residues in KPN_02808, the inhibition of the enzymatic activity by DTT might be due to the disruption of disulfide bonds that stabilize the protein [8]. The enzyme was not inhibited or sensitized by PMSF, suggesting that the KPN_02809 protein was not a serine protease. The results confirm the finding from our earlier computational analyses that suggest the hypothetical protein KPN_02809 belongs to the group of metalloprotease.

Effects of cation activators on the enzyme activity were also determined (Figure 8B). Calcium ion was shown to slightly activate the enzyme, while magnesium ion did not cause a significant change of the enzyme activity. The enzyme activity was inhibited by more than 60% in the presence of zinc, manganese, cuprum and cadmium ions. Interestingly, the recombinant enzyme showed catalytic activity without the need of additional cation such as zinc ion. It is possibly due to the conservation of bound divalent cation such as zinc ion in the purified recombinant KPN_02809 as previously reported for a mitochondrial peptidase [10]. To confirm that the zinc ion was preserved in the protein after purification, quantification of zinc ion on the purified protein was carried out according to the method of Bell *et al.* [17]. Results in Table 1 show that there were 1.2 molar of zinc ion in one molar of KPN_02809 protein and this confirms that the recombinant KPN_02809 contained one zinc ion in each of the protein molecule. The zinc binding of E172DKPN_02809 was not much affected. However, the molar ratio of zinc to H171SKPN_02809 mutant protein was only about 0.4, indicating the importance of the His171 in zinc binding of this protein. Previously, mutagenesis of the corresponding histidine and glutamate in HEXXH motif of *Clostridium histolyticum* ColH collagenase also showed that the histidine but not the glutamate was important as zinc ligand [18].

As shown in Figure 8B, the activity of KPN_02809 was inhibited by the addition of zinc ion, this is similar to other zinc metalloproteases, such as thermolysin [19] and carboxypeptidase A [20], which were also inhibited by an excess of zinc ion. Metalloproteases require a transition metal cations or alkaline earth cations for activity [16]. Zinc ion is an essential cofactor for the biological function of most metalloproteases. It directly participates in the catalytic activity or involves in maintaining the protein structure and stability [21]. The excess of zinc competitively inhibits the correct positioning of substrate into the pocket of active site [10]. In the presence of excess zinc, a second zinc ion can bind to the active site of the enzyme and causes distortion towards the tetrahedral coordination of the protein complex. This additional zinc ion assumed the position normally held by active water molecules and probably perturbed the substrate positioning and structural arrangement during catalysis reaction [19,20]. Thus, a decrease in enzyme activity by excess zinc was suggested to be due to the steric exclusion of the substrate from the active site.

## 3. Experimental Section

### 3.1. Homology Modeling of KPN_02809 Protein and Model Assessment

Preliminary analysis of the sequence of KPN_02809 was performed by using Uniprot [6]. Multiple sequence alignment was done by using MAFFT [22]. The template for structure prediction was chosen based on the result from the Phyre [12], Interproscan [13] and I-TASSER [23] analyses. MODELLER 9v8 [24] was used for model building. The zinc atom in our model was positioned based on the coordinate of zinc in the template structure using the “SET HETATM_IO = ON” command in MODELLER. 20 models were generated randomly and model with the best Discrete Optimized Potential Energy (DOPE) scoring was selected. Verification of the built model was done using PROCHECK [25].

### 3.2. Molecular Docking Simulation

ProBis [26] was used to detect the potential ligand’s- binding site on KPN_02809 model. Docking of selected protease inhibitors to the model structure was performed using Autodock 3.0.5 [27]. With the aid of Autodock Tool, Kollman-Amber united atom partial charges and solvation parameter were added to the built model of KPN_02809. Rotatable bonds were assigned for inhibitors while theirs partial charges were assigned with Gasteiger charges. All of the non-polar hydrogen from both the built protein model and inhibitors were merged. Grid map of 60 × 60 × 60 grid points and 0.375 Å spacing were generated using Autogrid3 and centered on the potential binding site. A total of 300 runs with 250 population size and root-mean square tolerance of 1.0 Å were set as the docking input parameter. The lowest docked energy of each conformation in the most populated cluster was selected.

### 3.3. Bacterial Strains, Growth and Culture Conditions

*Klebsiella pneumoniae* subsp. *pneumoniae* MGH 78578 (ATCC number 700721) was used for this study. The bacterial strain was routinely cultured in Luria-Bertani medium at 37 °C. *E. coli* XL1-Blue and BL21 (DE3) were used as the host strains for cloning and expression purposes. pGEX-RB vector [28], was used for the overexpression of the GST-tagged target protein in *E. coli* cell.

### 3.4. Total Genomic DNA and RNA Extractions from K. pneumoniae MGH 78578

Genomic DNA and total RNA were isolated from a 5 mL overnight culture of *K. pneumoniae* MGH 78578 using QIAmp DNA Mini Kit (Qiagen) and RNeasy Mini Kit (Qiagen), respectively. The integrity and size distribution of total purified RNA was visualized by ethidium bromide staining after electrophoresis on a 1% agarose gel.

### 3.5. Cloning and mutagenesis of K. pneumoniae ypfJ Open Reading Frame

The *ypfJ* ORF was PCR amplified from *K. pneumoniae* genomic DNA in a 50 μL reaction consisted of 10 × Thermopol buffer (New England Biolabs), 1 μM each of ypfJ-specific forward (5′-GAATTCCATATGCGCTGGCAAGGGCGTCGCG-3′) and reverse (5′-CGCGGATCCTTACAGCGCACTGCCGAAGGTATTG-3′) primers (underlined are the *Nde*I and *BamH*I recognition sequences for cloning), 5 mM dNTPs, 1 unit of Taq polymerase and 100 ng genomic DNA. The PCR was performed for 30 cycles at 95 °C for 30 s, 66 °C for 30 s and 72 °C for 60 s.

The PCR product was gel purified by Qiaquick gel extraction kit (Qiagen), digested with *Nde*I and *BamH*I (New England Biolabs) and ligated into a pGEX-RB precut with the same restriction enzymes. The resulting pGEX-RB-ypfJ was confirmed by sequencing. H171SKPN_02809 and E172DKPN_02809 mutant constructs were created by site directed mutagenesis of pGEX-RB-ypfJ according to method described previously [29]. Briefly, the first step involved PCR amplification of two separate products with overlapping ends by using mutagenesis primers and the ypfJ-specific primers (given above). In the second PCR step, the two overlapping fragments were fused by the gene-specific primers. The resulting PCR product was cut with *Nde*I/*Bam*HI and cloned into pGEX-RB vector to generate pGEX-RB-H171S-ypfJ and pGEX-RB-E172D-ypfJ. Primer sequences for

H171S mutation are; forward: 5′-GTACGTCATTGCTAGTGAAGTGGGTCATC-3′ and reverse: 5′-GATGACCCACTTCACTAGCAATGACGTAC-3′. Primer sequences for E172D mutation are; forward: 5′-GTCATTGCTCATGATGTGGGTCATCAC-3′ and reverse: 5′-GTGATGACCCACATC ATGAGCAATGAC-3′. The mutations were confirmed by sequencing.

### 3.6. Expression and Purification of Wild-Type and Mutant KPN_02809 Protein

For protein expression, the pGEX-RB-ypfJ, pGEX-RB-H171S-ypfJ and pGEX-RB-E172D-ypfJ plasmids were transformed into the *E. coli* BL21 (DE3) strain. The culture was grown in LB medium (with 100 μg/mL ampicillin) at 37 °C, 200 rpm to an OD_600 nm_ of 1.8. Subsequently, the expression of GST-tagged KPN_02809 was induced with 0.5 mM isopropyl thiogalactoside (IPTG). After the induction period, the cells were pelleted and re-suspended in 5 mL pre-cooled buffer (50 mM Tris-HCI, pH 7.5, 300 mM sodium chloride, 1% Trition X and 5 mM β-mercaptoethanol). The cells were then sonicated and centrifuged at 2000 × g for 20 min. The supernatant was mixed with the GST-binding resin 3 hours at 4 °C to allow binding. The resin was washed for several times with washing buffer (50 mM Tris (pH 7.5), 300 mM NaCl, 0.5% Triton X and 10% glycerol). After washing, the protein of interest was eluted from the GST-binding resin by thrombin cleavage and quantified by using Bradford reagent (Bio-Rad).

### 3.7. Reverse Transcription PCR of ypfJ Gene

RevertAid H Minus first strand cDNA synthesis kit (Fermentas) was used to synthesize the cDNA from the extracted total RNA. One microgram of total RNA was mixed with 0.2 μg random hexamer primer and DEPC-treated water. Subsequently, the mixture was preheated at 70 °C for 5 min, chilled on ice and followed by the addition of 4 μL of 5 × RT buffer, 1 mM of dNTP mix and 20 units of Ribolock Ribonuclease inhibitor. The mixture was incubated at 25 °C for 5 min followed by incubation at 37 °C for another 5 min. 200 units of Revertaid H Minus M-MuLVRT were added to a total volume of 20 μL. The mixture was then incubated at 42 °C for 1 h and heated at 70 °C for 10 min for the termination of the reverse transcription process. PCR was performed as described above except that the template was replaced with one μL of cDNA.

### 3.8. Casein Hydrolysis Assay

Enzymatic assays were performed with the QuantiCleave Protease Assay Kit (Pierce). Under the standard test condition, the enzymatic activity was measured at 25 °C, pH 8 at different reaction times, ranged from 15 until 90 min. The reaction was initiated by adding 20 μg of KPN_02809 hypothetical protein into the microplate well that contained 100 μg succinylated casein. After incubation, 50 μL of TNBSA solution were added to the well and incubated for additional 30 minutes. The absorbance at 450 nm was determined for every well and the proteolytic activity was represented by the change in absorbance (ΔA_450_), which was calculated by subtracting the absorbance of the blank from that of the corresponding casein well. Data from a triplicate experiment were analyzed with one way ANOVA using SPSS version 15.0. The level of significance was set at *p* ≤ 0.05.

### 3.9. Determination of pH and Temperature Optima for the Activity of Purified KPN_02809

20 μg of purified protein and 100 μg of succinylated casein were used per assay for the determination of optimum pH and temperature for KPN_02809 casein hydrolysis activity. The buffers used were 300 mM acetate buffer for pH 5.0 and 6.0, and 120 mM disodium hydrogen orthophosphate buffer for pH 7.4, 8.0 and 9.0. The effect of temperature on the protease activity of KPN_02809 was studied by incubating the standard reaction mixture at temperatures ranging from 20 °C to 40 °C for 60 min.

### 3.10. Effect of Various Inhibitors and Ions on the KPN_02809 Enzymatic Activity

The inhibitors used in this study are ethylenediaminetetraacetic acid disodium salt, EDTA (Univar Analytical Reagent), 1,4-dithiothreitol, DTT (Roche), phenylmethanesulfonyl fluoride, PMSF (Fluka Analytical) and 1,10-phenanthroline (Sigma Aldrich). One millimolar of the inhibitors was preincubated at room temperature for an hour with the hypothetical protein. The reaction was performed under the standard assay conditions. The relative activity was determined as a percentage of the activity in control samples (reaction without inhibitors). For study on the effect of ions on the enzymatic activity, 1 mM of each ion (Mg^2+^, Ca^2+^, Zn^2+^, Mn^2+^, Cu^2+^ and Cd^2+^) was added into the reaction mixture, incubated at 30 °C for 60 min. before adding the TNBSA and later incubated for another 30 minutes. The activity was then compared with the activity in control sample.

### 3.11. Quantification of Zinc Ion Content in the KPN_02809 Protein Molecule

The zinc ion quantification was carried out according to Bell *et al.* [17] by using a standard curve of absorbance at 500 nm *versus* zinc (II) ion concentration from 0 to 300 μM in 50 mM HEPES/KOH pH 7.5 containing 3% (v/v) perchloric acid. Each measurement was repeated for at least 3 times. The ratio of zinc to protein concentrations was then calculated.

## 4. Conclusions

In this work, a combination of protein sequence analysis, structural modeling and molecular docking simulation approaches were employed to probe the possible functions and characteristics of a hypothetical protein with unknown structure and biochemical properties. The catalytic behavior of this protein was subsequently investigated by first cloning the ORF of *ypfJ* gene that encodes this protein, followed by successful expression in *E. coli* and purification of the gene product. Characterization of the highly purified recombinant KPN_02809 confirmed the predictions made by computational analysis that this enzyme is a zinc metalloprotease. The recombinant KPN_02809 protein and its enzymatic properties obtained in this work could serve as the foundation for more structure function studies of this protein and investigation into its possible role in the pathogenesis of *K. pneumoniae*.

## Figures and Tables

**Table 1:** Quantification of Zinc ion in the KPN_02809 protein molecule.

Protein	Absorbance (500 nm)	Zinc concentration (μM)	Protein concentration (μM)	Ratio of zinc to protein concentration
BSA (negative control)	0.001	0	15.15	0.1
KPN_02809	0.456	149	124.60	1.2
E172DKPN_02809	0.167	61.85	62.50	1.0
H171SKPN_02809	0.073	27.04	62.50	0.4
